# Cloning and analysis of a bifunctional methyltransferase/restriction endonuclease TspGWI, the prototype of a *Thermus *sp. enzyme family

**DOI:** 10.1186/1471-2199-10-52

**Published:** 2009-05-29

**Authors:** Agnieszka Zylicz-Stachula, Janusz M Bujnicki, Piotr M Skowron

**Affiliations:** 1 Division of Theoretical Physical Chemistry, Department of Chemistry, University of Gdansk, Sobieskiego 18, 80-952 Gdansk, Poland; 2 Laboratory of Bioinformatics and Protein Engineering, International Institute of Molecular and Cell Biology in Warsaw, Ks. Trojdena 4, 02-109 Warsaw, and Institute of Molecular Biology and Biotechnology, Faculty of Biology, Adam Mickiewicz University, Umultowska 69, Poznan, Poland; 3 Division of Environmental Molecular Biotechnology, Department of Chemistry, University of Gdansk, Sobieskiego 18, 80-952 Gdansk, Poland

## Abstract

**Background:**

Restriction-modification systems are a diverse class of enzymes. They are classified into four major types: I, II, III and IV. We have previously proposed the existence of a *Thermus *sp. enzyme family, which belongs to type II restriction endonucleases (REases), however, it features also some characteristics of types I and III. Members include related thermophilic endonucleases: TspGWI, TaqII, TspDTI, and Tth111II.

**Results:**

Here we describe cloning, mutagenesis and analysis of the prototype TspGWI enzyme that recognises the 5'-ACGGA-3' site and cleaves 11/9 nt downstream. We cloned, expressed, and mutagenised the *tspgwi *gene and investigated the properties of its product, the bifunctional TspGWI restriction/modification enzyme. Since TspGWI does not cleave DNA completely, a cloning method was devised, based on amino acid sequencing of internal proteolytic fragments. The deduced amino acid sequence of the enzyme shares significant sequence similarity with another representative of the *Thermus *sp. family – TaqII. Interestingly, these enzymes recognise similar, yet different sequences in the DNA. Both enzymes cleave DNA at the same distance, but differ in their ability to cleave single sites and in the requirement of S-adenosylmethionine as an allosteric activator for cleavage. Both the restriction endonuclease (REase) and methyltransferase (MTase) activities of wild type (wt) TspGWI (either recombinant or isolated from *Thermus *sp.) are dependent on the presence of divalent cations.

**Conclusion:**

TspGWI is a bifunctional protein comprising a tandem arrangement of Type I-like domains; particularly noticeable is the central HsdM-like module comprising a helical domain and a highly conserved S-adenosylmethionine-binding/catalytic MTase domain, containing DPAVGTG and NPPY motifs. TspGWI also possesses an N-terminal PD-(D/E)XK nuclease domain related to the corresponding domains in HsdR subunits, but lacks the ATP-dependent translocase module of the HsdR subunit and the additional domains that are involved in subunit-subunit interactions in Type I systems. The MTase and REase activities of TspGWI are autonomous and can be uncoupled. Structurally and functionally, the TspGWI protomer appears to be a streamlined 'half' of a Type I enzyme.

## Background

Restriction-modification systems (RM) usually consist of at least two enzymatic activities: a restriction endonuclease (REase), which recognises and cleaves a specific DNA sequence, and a cognate DNA methyltransferase (MTase) that can methylate the same target site, thereby preventing the cleavage of host DNA by the REase [1]. On the basis of subunit composition, cofactor requirements, and the mode of recognition and cleavage, RM systems have been traditionally classified into three major types: I, II and III. Type IV has been re-introduced to describe a heterogeneous group of enzymes cleaving only modified DNA, with ill-defined sequence specificity [2]. Type I and Type III REases are complex multisubunit molecular machines that utilize ATP and perform either DNA modification or cleavage [3].

Type II REases are simpler: they recognise short, 4–8 bp, nucleotide sequences and cleave phosphodiester bonds in DNA within their recognition sites or at up to a distance of 20 bp from them [4]. In most cases, methylation and cleavage are divided between two separate enzymes. More than 3600 various REases that recognise more than 250 different nucleotide sequences have been described as members of this large and divergent class of enzymes [5]. Most of them have been isolated from mesophilic microorganisms and only a few from hyperthermophiles [6-8] and thermophiles, in particular from the genus *Thermus *[9-15].

In order to account for their structural and functional heterogeneity Type II REases have been divided into multiple subtypes, often overlapping with each other [2]. For example, a growing group of atypical Type II REases, represented by bifunctional enzymes with REase and MTase activities within a single polypeptide (subtype IIC), is a subclass of subtype IIS enzymes that recognise a specific DNA sequence and cleave outside it at a defined distance, within any sequence. Examples of this group include Eco57I [16,17], HaeIV [18], AloI [19], BseMII [20] and enzymes from the *Thermus *sp. family: TspGWI [recognition sequence: ACGGA (11/9)], TaqII [(GACCGA (11/9), CACCCA (11/9)], TspDTI [(ATGAA (11/9)] and Tth111II [(CAARCA (11/9)] [9,10].

In this paper we describe the cloning of the *tspgwi *gene based on degenerated primers PCR protocol, site-directed mutagenesis, expression and purification of recombinant and mutant proteins followed by comparative biochemical and genetic analysis. The constructed mutants are more suitable than native TspGWI in gene cloning methodology, as the cleavage and modification functions were separated into distinct, active proteins. TspGWI-like enzymes [9] are difficult to fit unambiguously into the conventional classification, as their unique structural and functional features can be found in REase sub-types IIA, IIC, IIS, IIF, IIH and type I.

## Results and discussion

### Sequencing, cloning and expression of the *tspgwi *gene

Neither the biochemical selection for methylation phenotype approach nor the "white-blue" screen for DNA damage/modification to yield recombinant clones were successful (not shown), due to TspGWI low activity and lack of complete cleavage of the plasmid DNA. We devised a cloning protocol for thermophilic REase without accompanying (separate) MTase, which includes two stages: (*i*) a gene nucleotide sequence determination starting from internal amino acid sequences of REase proteolytic fragments followed by various PCR methods using combination of degenerated and non-degenerated primers. The primers are designed using probability of codon usage from other closely related bacteria and (*ii*) direct in-frame insertion of amplified ORF into tightly temperature-regulated expression vector, taking advantage of low temperature cultivation at non-permissive conditions, which both prevent REase expression and suppresses activity of any leaking thermophilic REase.

**Figure 1:**
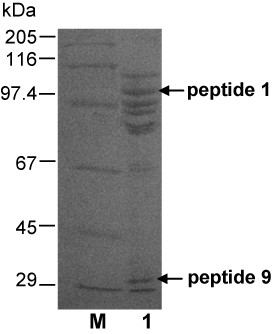
**Limited proteolytic digestion of TspGWI**. Purified native TspGWI was subjected to proteolytic digestion on immobilised TPCK-trypsin. Lane M, protein marker (Sigma), bands marked: 205 kDa, myosin; 116 kDa, β-galactosidase; 97.4 kDa, phosporylase b; 67 kDa, bovine serum albumin; 45 kDa, ovoalbumin; 29 kDa, carbonic anhydrase; lane 1, proteolysis products. Out of 10 polypeptides obtained, the two subjected to N-terminal sequencing are marked with horizontal arrows.

The *tspgwi *gene nucleotide sequence determination was initiated from very limited amino acid sequence information about the N-termini of native TspGWI proteolytic internal fragments. We were unable to determine the amino acid sequence of the intact protein, thus homogeneous TspGWI preparation was subjected to limited proteolysis, yielding 3 major bands and more than 10 minor bands, as determined by SDS/PAGE (Figure 1; see Additional file 1). The immobilised TPCK-trypsin was readily removed by centrifugation; hence, the resulting bands were derivatives of TspGWI protein only. Two of the predominant bands of approximate sizes 30 kDa and 100 kDa were subjected to N-terminal sequencing (Figure 1, lane 2). Based on the obtained short 19- and 10- amino acid sequences GPIGGGGSPEAQLVPLITR and EFFTERLAQE, sets of non-degenerated and degenerated primers, forming alternative pairs, were designed. The primers were designed arbitrarily as based on back-translated amino acid sequence using codon usage data from ORFs of *Thermus *sp. genes available in GenBank. The high GC content of *Thermus *genes was also considered. Sets of primer pairs used were either with low degeneracy and non-degenerated, containing incorporated codons of the assumed highest probability to exist in *Thermus *sp.

**Figure 2:**
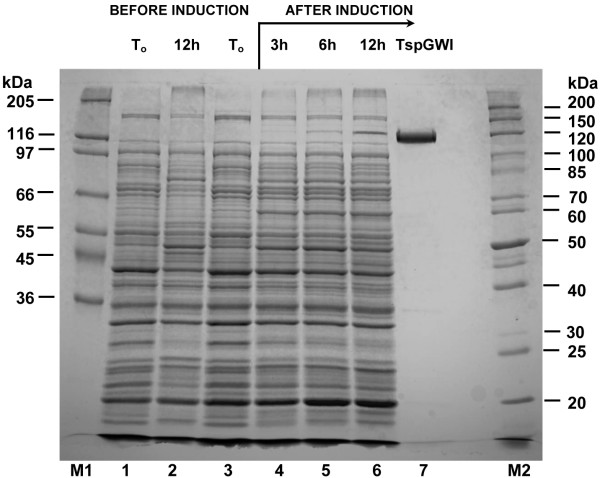
**SDS/PAGE analysis of the purified recombinant wt TspGWI endonuclease**. Lane M1, high protein marker (Sigma), bands marked: 205 kDa, myosin, 116 kDa, β-galactosidase, 97 kDa, phosporylase b; 66 kDa, bovine serum albumin; 55 kDa, glutamic dehydrogenase, 45 kDa, ovoalbumin; 36 kDa, glyceraldehyde-3-phosphate dehydrogenase; lane 1, control culture – crude lysate from *E. coli *expressing the cloned *tspgwi *gene, without induction (OD_600 _= 0.7); lane 2, control culture – crude lysate from *E. coli *expressing the cloned *tspgwi *gene without induction, after 12 h of cultivation; lane 3, crude lysate from *E. coli *expressing the cloned *tspgwi *gene, before induction (OD_600 _= 0.7); lane 4, crude lysate from *E. coli *expressing the cloned *tspgwi *gene, 3 h after induction; lane 5, crude lysate from *E. coli *expressing the cloned *tspgwi *gene, 6 h after induction; lane 6, crude lysate from *E. coli *expressing the cloned *tspgwi *gene, 12 h after induction; lane 7, purified, homogeneous recombinant wt TspGWI protein; lane M2, protein marker (Fermentas), bands marked: 200 kDa, 150 kDa, 120 kDa, 100 kDa, 85 kDa, 70 kDa, 60 kDa, 50 kDa, 40 kDa, 30 kDa, 25 kDa, 20 kDa.

One such pair yielded a PCR fragment containing an internal portion of the *tspgwi *gene. Combination of PCR with degenerated primers using *Thermus *sp. GW genomic DNA as a template as well as casette-ligation-PCR using pBR322 DNA as the known sequence donor for non-degenerated primers, yielded a sequence of 3574 bp contig with the complete *tspgwi *gene. Each contig strand was sequenced; in addition, regions containing discrepancies were sequenced several times under different conditions. The *tspgwi *gene ORF coding for the REase-MTase bifunctional protein is 3291 bp in length coding for the 1097 amino acid polypeptide [GenBank:EF095488, ABO26710]. The calculated molecular weight of the TspGWI is 120 202 Da, which perfectly matches data from SDS/PAGE (Figure 2) and size exclusion chromatography of the native protein [9,10]. The calculated isoelectric point of recombinant TspGWI is 6.58 (DNASIS MAX 1.1 software), very similar to calculated isoelectric point of recombinant TspDTI of 6.68 (manuscript in preparation).

No sequence similarity to any MTase or DNA-binding protein was found in the ORF flanking regions. The ORF begins with the ATG start codon and contains 2 putative upstream Ribosome Binding Sites (RBSs): -11 5'-AGG and -15 5'-AGGA. The ORF is extremely GC rich: 74.81%. To ensure that no errors are incorpoated during *tspgwi *gene amplification, all the PCR reactions were performed with a proofreading blend of Taq and Pfu DNA polymerases. Sequencing was repeated three times for each strand, both directly for the independent PCR products and for the cloned the gene fragments.

Native TspGWI purification was reported by us previously [10]. Since TspGWI activity was hardly noticeable in its natural host *Thermus *GW, the development of an extensive isolation procedure was essential, which included: polyethyleneimine precipitation of nucleic acids, ammonium sulfate fractionations followed by five chromatographic steps and size-exclusion chromatography [10]. To the contrary, recombinant TspGWI and its mutants were purified to homogeneity using much simplified procedure with the TspGWI protein expressed in *E. coli*. The protocol included heat-denaturing *E. coli *proteins, polyethyleneimine precipitation of nucleic acids, ammonium sulfate fractionations, followed by two chromatographic steps.

Interestingly, in spite of the cloning in the presence of a strong P_R _promoter, the protein becomes detectable in induced cells only after 3 h of growth under non-permissive conditions, and keeps accumulating until the late stationary phase, even after 12 h cultivation at 42°C (Figure 2). This is probably due to the combination of the following factors: (*i*) GC rich ORF sequence distant from *E. coli *optimum codon usage, (*ii*) slow transcription of the extremely GC-rich *tspgwi *gene, (*iii*) the presence of numerous putative hairpin structures within the gene and (*iv*) the very large size of the protein to be translated. Nevertheless, optimisation of expression culture growth/induction conditions yielded adequate amounts of TspGWI (about 31 mg of the protein per litre of bacterial culture). It was also observed that the initially homogenous protein, after prolonged storage at -20°C, undergoes minor degradation (not shown).

### Bioinformatic analyses of TspGWI: prediction of domains and functional motifs

**Figure 3:**
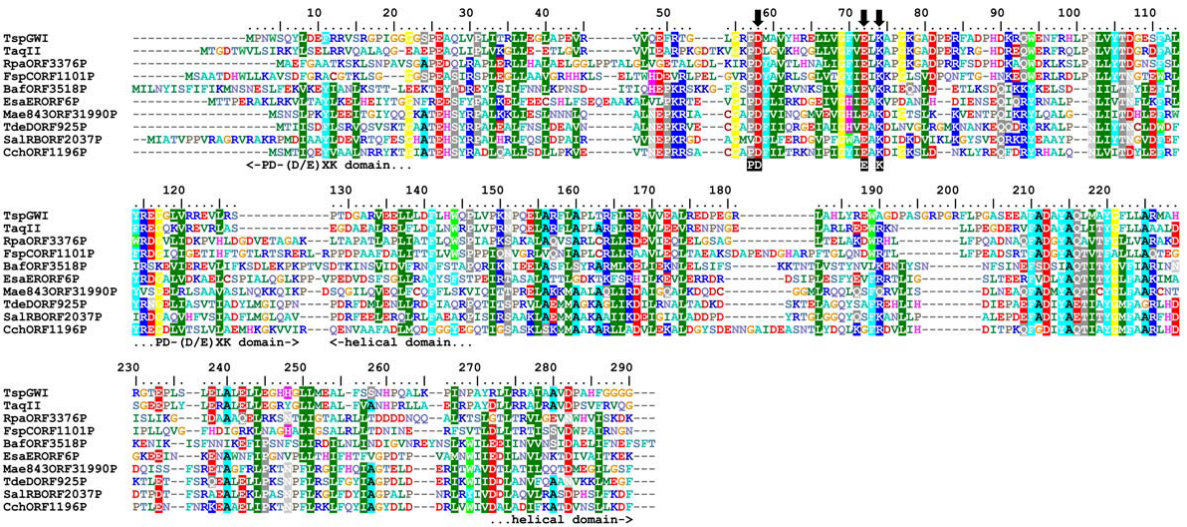
**Sequence alignment between TspGWI and its close homologues in REBASE (BLAST E-value < 1E-40)**. The PD-(D/E)XK domain with the nuclease active site and a helical domain. The residues subjected to site-directed mutagenesis are indicated by arrows.

**Figure 4:**
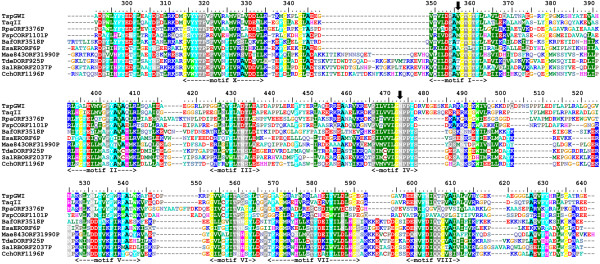
**Sequence alignment between TspGWI and its close homologues in REBASE (BLAST E-value < 1E-40)**. The RFM domain with the MTase active site. The residues subjected to site-directed mutagenesis are indicated by arrows.

**Figure 5:**
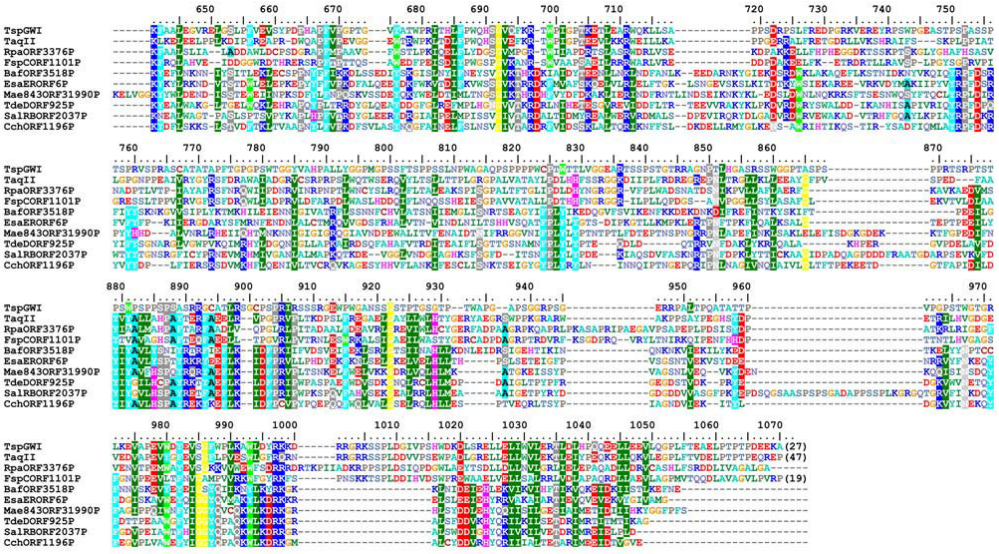
**Sequence alignment between TspGWI and its close homologues in REBASE (BLAST E-value < 1E-40)**. The predicted DNA target-recognition domain (TRD).

Isolation and sequencing of the *tspgwi *gene enabled detailed analysis of its amino acid sequence. Searches of REase sequences deposited in REBASE revealed an overall similarity to a number of genuine and putative Type IIC enzymes, including the previously characterised nuclease TaqII [21] (Figure 3, 4, 5). Two other members of the *Thermus *family, i.e. TspDTI (GenBank:EF095489, ABO26711] and Tth111II [GenBank:AY726624, AAU21502], showed no significant sequence similarity to TspGWI and TaqII in pairwise comparisons and were thus excluded from the alignment. Further bioinformatic analyses (see Methods) revealed the expected significant similarity of the central region of TspGWI (aa ~320–620) to the catalytic domains of known DNA:m^6^A MTases. No significant similarity of the terminal regions (aa 1–320 and 660–1097) to any protein family was observed in simple sequence searches. Nonetheless, multiple sequence alignment of TspGWI homologues revealed the presence of a candidate PD-(D/E)XK motif (Figure 3), resembling the active site of many REases and other nucleases [22]. We carried out a protein fold-recognition (FR) analysis (see Methods) to confirm this preliminary prediction for the N-terminal region, as well as to identify potential homologues and predict the structure for the C-terminal region. Since the FR method is designed to identify remote homology and predict structure for domain-size sequence fragments (20–500 aa), the TspGWI sequence was split into a series of overlapping segments and submitted to the GeneSilico metaserver [23].

The FR analysis of the TspGWI sequence identified the structures of a Type I DNA:m^6^A MTase M. EcoKI HsdM subunit (code 2ar0 in Protein Data Bank [24]), a putative HsdM subunit of an experimentally uncharacterised RM system BthVORF4518P (code 2okc in Protein Data Bank), and Type II DNA:m^6^A MTase M. TaqI (code 1g38 in Protein Data Bank) as the best structural templates for the central region comprising residues 130–660 of TspGWI (PCONS score 3.147 for M. EcoKI, 3.0995 for M. BthVORF4518P, and 2.5563 for M. TaqI, with scores > 1 indicating statistical significance). Interestingly, the FR alignments for this region revealed not only the S-adenosylmethionine (AdoMet)-binding/catalytic MTase domain (residues ~320–660 in TspGWI), but also a helical domain characteristic of the N-terminus of the HsdM subunit (residues ~130–320 in TspGWI). For the N-terminal region of TspGWI (residues ~1–130) the FR methods also supported the prediction of the PD-(D/E)XK domain, albeit with scores below the level of significance (PCONS score 0.5338 for the Holliday junction resolvase; 1hh1 in Protein Data Bank). The closest match on the sequence level (reported with a significant value of 135.67) by the HHSEARCH method for profile-profile searches was the N-terminal PD-(D/E)XK domain of the HsdR subunit from Type I RM systems [25]. This prediction made for the N-terminal domain of TspGWI further supports the classification of the catalytic domain of TaqII-related enzymes as a member of the PD-(D/E)XK fold [26].

FR methods failed to report any significant matches to known structures for the C-terminal part of the TspGWI sequence (data not shown). Interestingly, the C-terminal region was predicted to be almost entirely disordered, with only a short segment comprising residues 960–1060 predicted to form a stable helical secondary structure. This segment (but not residues 660–960 that are predicted to be disordered) shows sequence similarity to the C-terminus of TaqII. We found no similarity of this region to target recognition domains (TRD) of any MTase family; nevertheless, we speculate that it might play an analogous role.

**Figure 6:**
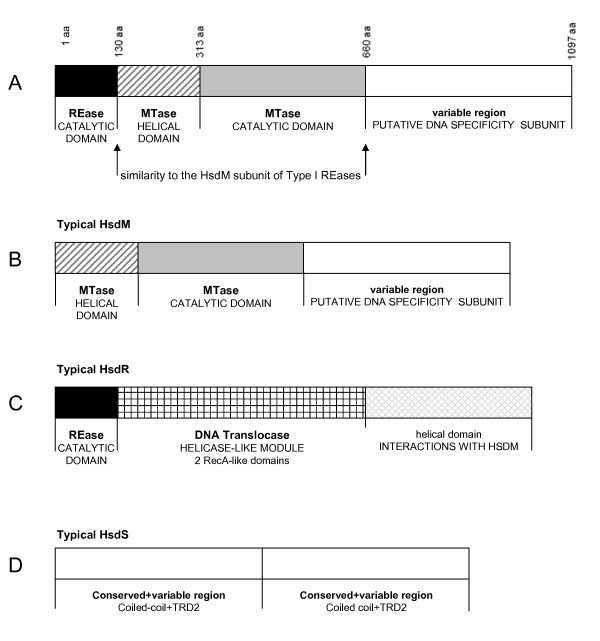
**Schematic organization of domains of bifunctional TspGWI enzyme**. (**A**) TspGWI subunits organization. Black block – catalytic domain, diagonal lines – MTase domain, grey block – MTase helical domain; white block – variable region, putative DNA specificity subunit. (**B**) Typical HsdM subunit organization. (**C**) Typical HsdR subunit organization. (**D**) Typical HsdS subunit organization.

Altogether, the results of our FR analysis suggest that Type II enzyme TspGWI is a fusion protein comprising a tandem arrangement of Type I-like domains: a PD-(D/E)XK nuclease domain related to the corresponding domains in HsdR subunits, a HsdM-like module comprising a helical domain and a highly conserved AdoMet-binding/catalytic MTase domain, and a C-terminal extension, presumably responsible for DNA sequence recognition (Figure 6). On the other hand, TspGWI lacks other domains characteristic of Type I RM enzymes, including the ATP-dependent translocase module of the HsdR subunit and C-terminal domains of either HsdM or HsdR, which are important for interactions between Type I subunits [25,27]. This domain organization resembles the structure of the recently analysed MmeI enzyme [28], to which TspGWI exhibits only very limited sequence similarity, restricted primarily to the central MTase domain (data not shown).

### Enzymatic properties of TspGWI

**Figure 7:**
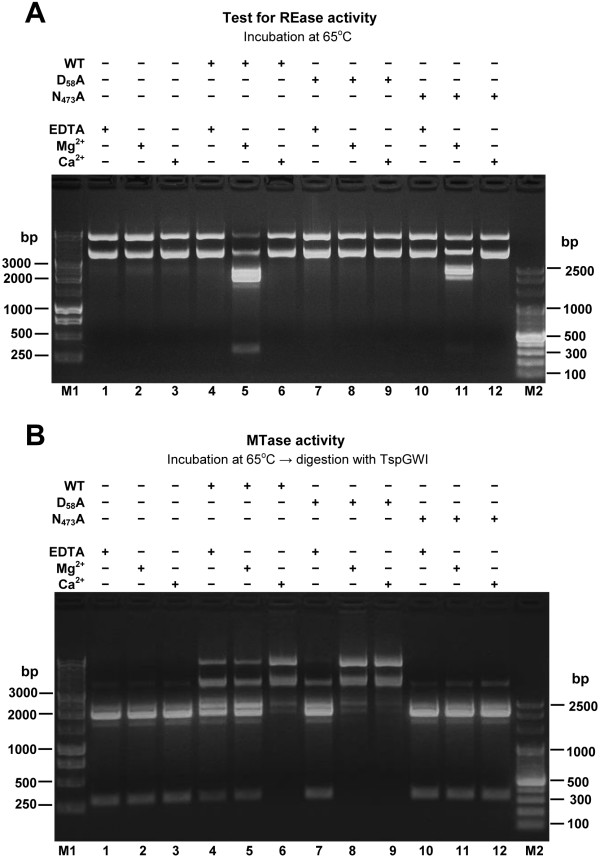
**Bifunctionality of the TspGWI: restriction and methylation activities of wt enzyme and selected mutants in the presence of divalent cations**. Samples of 2 μg pBR322 DNA were incubated with TspGWI protein in the TspGWI reaction buffer (50 mM Tris-HCl 8.0, 10 mM DDT) supplemented with AdoMet, in the presence/absence of metal ions (Mg^2+^, Ca^2+^) or EDTA. Proteins were removed by proteinase K digestion, DNA was purified and every sample was divided into two equal parts. (**A**) Effect of Mg^2+^, Ca^2+ ^on DNA restriction by TspGWI and its mutant variants. Lane M1, 1 kb DNA ladder (selected bands marked); lane 1 (-TspGWI; +EDTA), control 1 μg pBR322 DNA incubated in the reaction buffer devoid of Mg^2+ ^and supplemented with 10 mM EDTA, no enzyme; lane 2 (-TspGWI; +Mg^2+^), Mg^2+^present, no enzyme; lane 3 (-TspGWI; +Ca^2+^), Ca^2+^present, no enzyme; lane 4 (+TspGWI; +EDTA), 2.5 μg (30 u) of native TspGWI, Mg^2+ ^absent, supplemented with 10 mM EDTA; lane 5 (+TspGWI; +Mg^2+^), 2.5 μg of TspGWI, Mg^2+ ^present; lane 6 (+TspGWI; +Ca^2+^), 2.5 μg of TspGWI, Ca^2+ ^present; lane 7 (+D_58_A; +EDTA), 2.5 μg TspGWI mutant D_58_A, Mg^2+ ^absent, supplemented with 10 mM EDTA; lane 8 (+D_58_A; +Mg^2+^), 2.5 μg TspGWI mutant D_58_A, Mg^2+ ^present; lane 9 (+D_58_A; +Ca^2+^), 2.5 μg TspGWI mutant D_58_A, Ca^2+ ^present; lane 10 (+N_473_A; +EDTA), 2.5 μg TspGWI mutant N_473_A, Mg^2+ ^present, supplemented with 10 mM EDTA; lane 11 (+N_473_A; +Mg^2+^), 2.5 μg TspGWI mutant N_473_A, Mg^2+ ^present; lane 12 (+N_473_A; +Ca^2+^), 2.5 μg TspGWI mutant N_473_A, Ca^2+ ^present; lane M2, 100 bp DNA ladder (selected bands marked). (**B**) Effect of Mg^2+^, Ca^2+ ^on DNA methylation by TspGWI and selected mutant variants. Lane M1, 1 kb DNA ladder (selected bands marked). Lanes 1–12: all samples were treated as described above in (A), DNA purified and subjected to digestion by 3 units of TspGWI for 1 h at 65°C in the TspGWI REase buffer (50 mM Tris-HCl pH 8.0, 10 mM MgCl_2_, 10 mM DTT); lane M2, 100 bp DNA ladder (selected bands marked).

**Figure 8:**
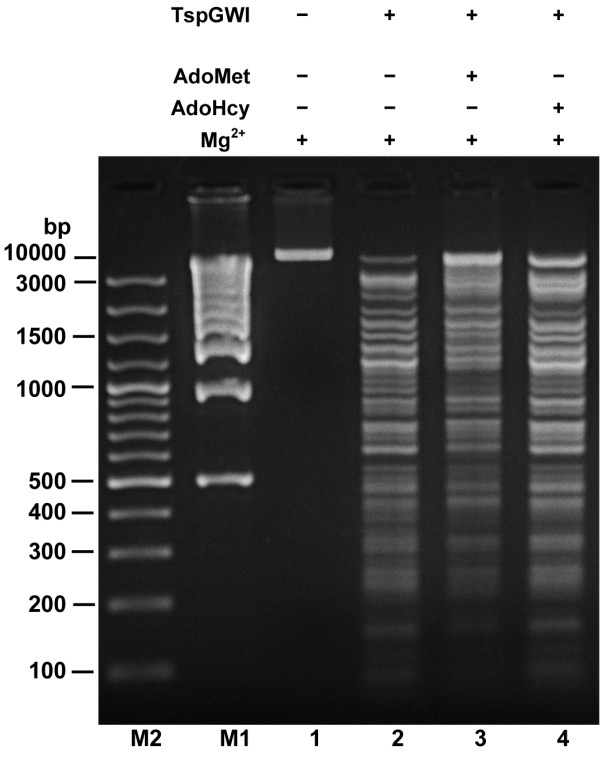
**Effect of AdoMet and AdoHcy on the TspGWI restriction of DNA**. Lane M1, 1 kb ladder (selected bands marked); lane M2, 100 bp ladder (selected bands marked); lane 1 (-TspGWI; +Mg^2+^; -AdoMet; – AdoHcy), control 1 μg of λ DNA; lane 2 (+TspGWI; +Mg^2+^;-AdoMet; – AdoHcy) λ DNA incubated with 1 u of TspGWI REase in the reaction mixture with Mg^2+ ^and without AdoMet; lane 3 (+TspGWI; +Mg^2+^; +AdoMet; -AdoHcy), λ DNA incubated with 1 u of TspGWI REase in the reaction mixture with Mg^2+ ^and with AdoMet; lane 4 (+TspGWI; +Mg^2+^;-AdoMet; + AdoHcy), λ DNA incubated with 1 u of TspGWI REase in the reaction mixture with Mg^2+ ^and with AdoHcy.

Purified preparation of native TspGWI protein was used for the detailed study of biochemical properties, reaction conditions and various cofactors influencing either DNA cleavage or the methylation activity of the enzyme. The apparent molecular mass of the wt recombinant protein under denaturing conditions was found to be 122 kDa, adequately to the molecular mass of TspGWI isolated from *Thermus *sp. GW [10]. Comparative assay of activities of both enzymes revealed no difference (not shown). Control mock-purification from *E. coli *did not show any of the described activities (not shown). Wt recombinant protein was also subjected to analytical gel filtration in a buffer with a composition close to the physiological, containing 3 mM MgCl_2 _(in the absence of DNA). The experiment revealed that the native endonuclease molecular mass is 110–130 kDa, indicating that under physiological conditions the protein exists as a monomer [9]. The temperature activity range extends from 42°C to 89°C (10% or more activity), with the maximum observed at 60–70°C. Incubation at 37°C resulted in 5% activity only (not shown). TspGWI is inactivated by 10 min incubation at 89°C and pH greatly affects activity, which drops sharply below pH 6.0 and above 9.5 (as measured at reaction temperature), with the maximum at 6.7–8.3 (not shown). The optimal ionic strength is below 25 mM KCl; at 50 mM only 50% of activity remains (not shown). The optimum restriction reaction conditions for TspGWI endonuclease *in vitro *are in 50 mM Tris-HCl pH 7.2 at 65°C, 10 mM MgCl_2_, 10 mM DTT. TspGWI maintains the absolute requirement for Mg^2+ ^for cleavage activity, with the optimum between 5 and 30 mM (Figure 7A). Mg^2+ ^ions can be effectively substituted with Mn^2+^, Fe^2+ ^and partially with Co^2+ ^ions (not shown). Remarkably, Ca^2+ ^ions do not support TspGWI cleavage activity, while in contrast, they stimulate methylation activity of the enzyme (Figure 7B). No difference was observed in response to divalent cations by both recombinant and native wt TspGWI (not shown). Unlike most of the other bifunctional Type IIC REases/MTases [16,29-34] 100 μM AdoMet slightly inhibits the REase activity of TspGWI enzyme (Figure 8, lane 3), while S-adenosylhomocysteine (AdoHcy) has essentially no effect on lambda DNA cleavage (Figure 8, lane 4). The restriction activity of TspGWI is neither inhibited nor stimulated by spermidine or ATP (not shown). As AdoMet is a co-substrate for the TspGWI MTase activity, it indirectly promotes DNA modification rather than cleavage. The presence of refractory sites, observed *in vitro *without AdoMet in the reaction buffer, could alternatively be explained by partial DNA methylation due to certain enzyme-bound AdoMet carry over during purification. However, since eight purification steps were used [10], this possibility is rather remote. In addition, partial protection should be uniform among different recognition sites, instead of observed site preference.

A recombinant TspGWI gene alone can be cloned into *E. coli *without an additional MTase (which thus far has not been found) and stably maintained at 28°C. A much reduced restriction activity < 5% at low temperature and the presence of cellular AdoMet and Ca^2+ ^appears to favour methylation *in vivo*. Taken together, the AdoMet and Ca^2+ ^effects as well as apparent preference for the opposite arrangement of recognition sites may provide an effective mechanism for controlling undesired restriction *in vivo*, thus removing the necessity for the existence of a separate TspGWI MTase. Another conclusion from the above results is that DNA methylation and DNA cleavage events occur by different mechanisms, in parallel and essentially independently. As a result, some of the target sites become protected, even under conditions favouring cleavage. Such concurrent methylation and restriction activities seem to resemble the behaviour of another bifunctional Type IIC enzyme AloI [19].

To further verify the marked independence of cleavage and methylation as well as to define the functional motifs within TspGWI (described in more detail in the following section), a series of amino acid replacements by site-directed mutagenesis was performed on TspGWI. We targeted three residues in the catalytic motif "PD-(D/E)XK" of the nuclease domain (D_58_, E_72_, K_74_), and two residues in the MTase domain of TspGWI: in the AdoMet-binding motif I "DPAVGTG" (V_356_) and in the main MTase catalytic motif IV: "NPPY" (N_473_). All the mutant proteins used were of high purity, isolated using simplified recombinant TspGWI protocol and their activity *in vitro *was analysed (Table 1, 2). Some of the mutants lost their REase activity, while maintaining the wild type (wt) level of methylation; others lost their MTase activity while retaining the ability to cut DNA, although at reduced efficiency (Figure 7A, B, lanes 7–12; Table 1, 2). The MTase activities of both the wt (native and recombinant) TspGWI and mutant proteins were dependent on the presence of divalent cations (Figure 7A, B, lanes 4–12; Table 1, 2). Further experiments are in progress to evaluate complex effect of Ca^2+ ^and Mg^2+^.

**Table 1:** Effect of divalent cations on the enzymatic activities of wt TspGWI and selected mutants of it.

**TspGWI variant**	**No divalent cation**	**Mg**^2+^	**Ca**^2+^	**Activity**
WT	__	+++	__	REase

WT	+	+	+++	MTase

D_58_A	__	__	__	REase

D_58_A	__	+++	+++	MTase

N_473_A	__	+	__	REase

N_473_A	__	__	__	MTase

**Table 2:** Methylation and restriction activities of TspGWI mutants.

**Mutation**	**REase activity**	**MTase activity**
WT	+++	+++

D_58_A	__	+++

D_58_R	__	+++

E_72_A	__	+++

E_72_D	+++	+++

E_72_L	__	+++

E_72_N	__	+++

E_72_R	__	+++

E_72_S	__	+++

K_74_A	__	+++

K_74_G	__	+++

K_74_P	__	+++

K_74_R	__	+++

V_356_M	+	+++

N_473_A	+	__

V_356_M/N_473_A	__	__

### DNA substrate preference of TspGWI

**Figure 9:**
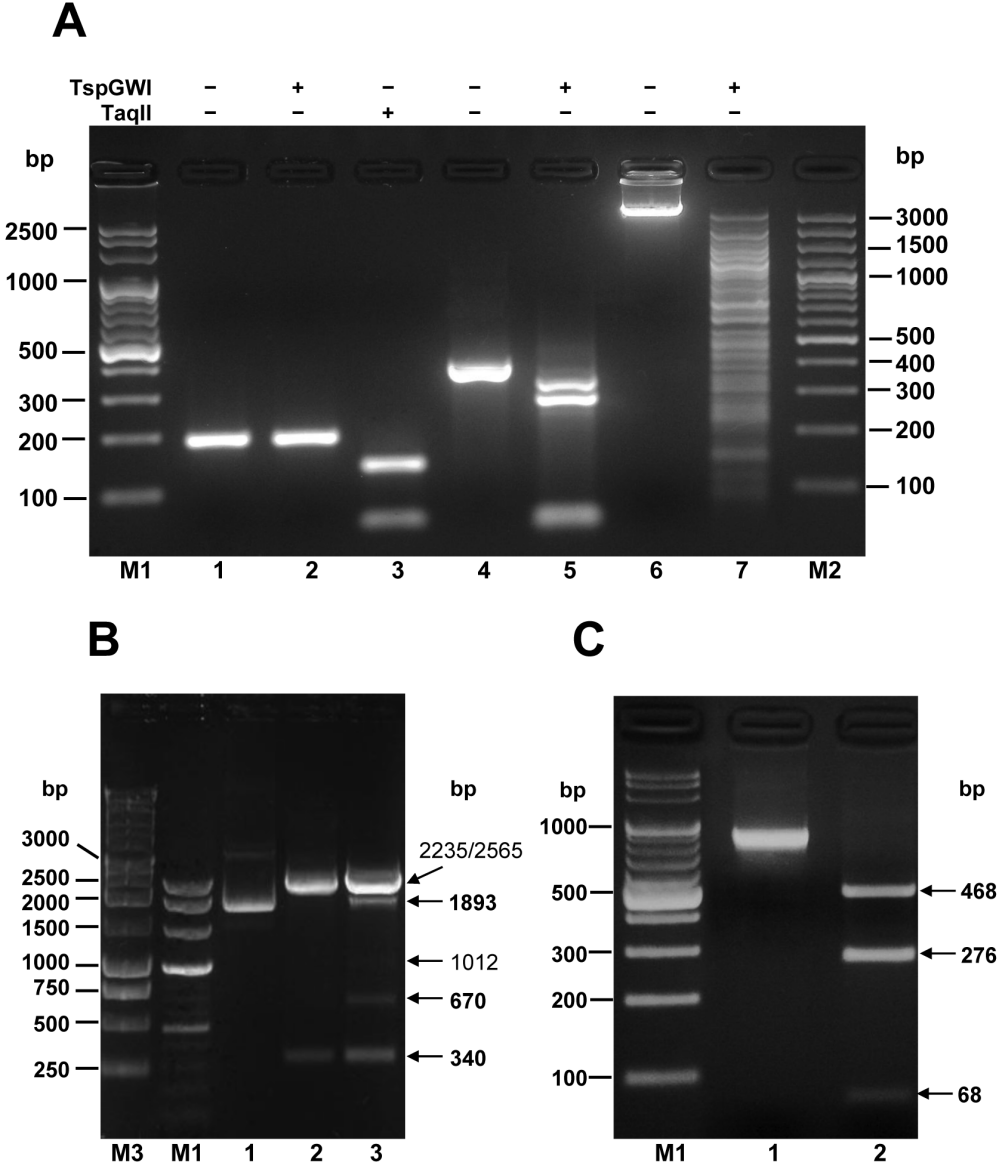
**TspGWI DNA substrate preferences**. (**A**) Resistance of DNA substrate with a single target site to TspGWI cleavage. A PCR fragment of 200 bp was generated, which contains unique recognition sequences for two *Thermus *sp. (sub)family enzymes: TspGWI and TaqII. Lane M1, 100 bp ladder (selected bands marked); lane 1, undigested 200 bp fragment; lane 2, digestion of the 200 bp PCR fragment with TspGWI; lane 3, digestion of the 200 bp PCR fragment with TaqII; lane 4, undigested 390 bp fragment (double sites; →←); lane 5, digestion of the 390 bp PCR fragment (double sites; →←) with TspGWI; lane 6, undigested bacteriophage lambda DNA; lane 7, digestion of bacteriophage lambda DNA with TspGWI; lane M2, 100 bp DNA ladder (selected bands marked). (**B**) Restoration of cleavability of the 200 bp PCR fragment by cloning into pUC19. The PCR fragment refractory to TspGWI cleavage was cloned into pUC19 (yielding pUC19-200), containing two existing TspGWI sites. The complete digestion pattern includes 340, 670 and 1893 bp restriction fragments (marked in bold). The partial digestion pattern (all 3 sites) includes 340, 670, 1010, 1893, 2232 and 2562 bp. The fragments obtained are marked with horizontal arrows. Lane M3, 1 kb ladder (selected bands marked); lane M2, 100 bp ladder; lane 1, control undigested pUC19 DNA; lane 2, pUC19 DNA digested with 3 u TspGWI; lane 3, pUC19-200 digested with 3 u TspGWI. **(C) **Susceptibility of DNA substrate with double divergent sites to TspGWI cleavage. A PCR fragment of 816 bp was generated, which contains two divergent recognition sequences TspGWI, separated by 40 bp spacer DNA. Lane M, 100 bp DNA ladder (selected bands marked); lane 1, undigested 816 bp PCR substrate; lane 2, digestion of the 816 bp DNA with 3 u TspGWI.

Under the variety of conditions initially tested no complete TspGWI digestion was obtained; instead a stable partial cleavage pattern was observed. It is probably due to the preferential site cleavage in various DNA substrates. Among them pUC19 was found to be the most efficiently cleaved supercoiled DNA substrate [10]. However, even in the case of pUC19 small fraction of molecules remained uncut, as determined by agarose gel electrophoresis of overloaded digestion samples and by transformation assay (not shown). The cleavage depends on the substrate used: it varies from the near complete digestion of two TspGWI sites in pUC19 DNA (Figure 9B, lane 2) to the trace cleavage of a subset of refractive sites in pBR322 and pACYC184 plasmids [10]. The refractory site phenomenon was preliminary evaluated here using two PCR substrates, containing either single (200 bp) or double (816 bp) TspGWI recognition sites. On a 200 bp PCR fragment containing a single recognition site for TspGWI and TaqII, no detectable cleavage was observed in the case of TspGWI (Figure 9A, lane 2), while closely related TaqII exhibits complete digestion (Figure 9A, lane 3). However, when the 200 bp fragment is cloned into pUC19 plasmid (pUC19-200PCR), the TspGWI site becomes cleavable, as evident from the appearance of predicted 670 bp and 1893 bp bands (Figure 9B, lane 3). The 816 bp PCR fragment contained two divergent TspGWI recognition sites, separated by a 40 bp spacer, which may allow two TspGWI molecules to bind simultaneously both recognition sequences located on the same face of DNA, without steric interference, while being close enough to allow for protein-protein interaction. It turned out to be the most completely cleaved substrate among those we tested (Figure 9C, lane 2). Further experiments are in progress to carefully evaluate the observed phenomenon, as the presence of two sites on the same DNA molecule may be an intrinsic part of the TspGWI scission mechanism.

The requirement for the presence of two recognition sites of opposite orientation for DNA cleavage is a characteristic feature of Type III REases [35]. Thus, the TspGWI-like enzymes (i.e. the *Thermus *sp. family defined earlier [9]) show a mixture of features characteristic of type I, II, or III RM systems. Type III enzymes methylate only one strand, regardless of the number and orientation of cognate sites, while still providing adequate protection against cleavage. Within type II (subtype IIS), BspMI is the one REase described to date that strongly prefers a tandem, head-to-tail orientation of double sites [36]. Another subtype IIS endonuclease FokI, has a preference towards a pair of sites with a tandem head-to-tail orientation [37]. The divergent orientations of the recognition sites may ensure cleavage of both sequences, as was proposed for type IIS REases BsgI, BpMI and Acc36I [38], which are, however, rather remotely related to TspGWI (in the sense of amino acid sequence similarity). Nevertheless, these enzymes retain the ability to cut single sites, although with a reduced efficiency compared to the tandem sites. TspGWI is unable to cut a single site, unlike BsgI, BpMI, Acc36I and the closely related TaqII REase. It is intriguing that TaqII REase, despite the very high amino acid sequence similarity to TspGWI (Figure 3, 4, 5) and in spite of numerous biochemical similarities [9] cleaves single sites efficiently. The ability to cleave a single recognition sequence may be correlated with the existence of REase activity stimulation by AdoMet. Although this cofactor has no effect on TspGWI REase activity, it does stimulate TaqII REase approximately 2–3 fold (manuscript in preparation). As we have pointed out previously [9], the new *Thermus *sp. (sub)family including: TspGWI, TaqII, TspDTI and Tth111II, is internally diversified. Even though these enzymes exhibit striking similarities in protein size, quaternary structure, recognition sequences, cleavage distances and related origin from various *Thermus *strains, they display marked differences in their response to AdoMet and to the arrangement requirements of different recognition sites. Interestingly, this group of enzymes seems to be also heterogenous at the level of amino acid sequence similarity, as TspGWI and TaqII seem to be closely related to each other, but only remotely to the two other enzymes TspDTI and Tth111II. This latter pair of enzymes appear to be closely related to each other, but exhibit a different pattern of similarities compared to the TspGWI-TaqII pair. However, all four enzymes share significant biochemical similarities, including related recognition and cleavage sequences, very large molecular weights of around 120 kDa, monomeric protein organization [10] and are negatively charged with calculated isoelectric points of 6.58 (TspGWI), 6.68 (TspDTI), 5.40 (TaqII) and 6.60 (Tth111II) (manuscript in preparation).

Although TspGWI is not particularly closely related to any known subtype IIB REase [26], the similarity of the domain structure and the mode of action of these enzymes (all belong to Type IIC) suggests that they have evolved from a common ancestor. Thus, type IIB enzymes forming tight dimers and TaqII-like enzymes that hardly dimerise if at all, may represent two extremes of an evolutionary landscape, where different enzymes have specialised towards either separation or pairing of recognition sites by developing subunit-subunit interactions of different strength. In this classification, TspGWI would be located "between" Type IIB enzymes and TaqII.

### Endonucleolytic activity of TspGWI wt and the mutant proteins

Site-directed mutagenesis was used to test whether the amino acids predicted by bioinformatics were essential for the MTase and REase catalytic activity of the TspGWI enzyme. To this end, variants of the TspGWI protein with substitutions in the putative PD-(D/E)xK domain and in the MTase domain were constructed by saturation site-directed mutagenesis. The mutant proteins were purified as described for recombinant TspGWI protein and subjected to *in vitro *biochemical experiments. To calibrate the protein concentrations, each variant was assayed, then diluted with the storage buffer to achieve equal protein concentration in every assay. The purity and concentration of the diluted proteins were further assessed by poliacrylamide gel electrophoresis (not shown). The cleavage reaction was performed at 65°C for one hour and overnight. Mutants in the PD-(D/E)XK domain D_58_A, D_58_R, E_72_A, E_72_L, E_72_N, E_72_R, E_72_S, K_74_A, K_74_G, K_74_P, K_74_R did not show any DNA cleavage activity under optimal buffer conditions at a concentration of 73 μg/ml, the equivalent of 27 u wt TspGWI per reaction. Higher enzyme concentrations could not be tested, as the specific activity of wt TspGWI REase is relatively low (12 400 u/mg), and increasing the amount of protein per reaction caused inhibition because of its non-specific binding to the substrate DNA. Only one mutant variant E_72_D retained its REase activity – its cleavage rate was even slightly higher than that of the wt protein. Thus, conservative substitution of aspartic acid for glutamic acid in the (D/E) site keeps the PD-(D/E)XK motif of TspGWI fully functional. It also confirms the key role of acidic amino acid in position 72 in catalysis. Since preferential cleavage of substrate DNA by native TspGWI was observed at concentrations as low as 0.35 μg/ml (0.13 u/reaction), the decrease in REase activity of TspGWI mutants was estimated to be more than 200-fold, thus confirming the predicted role of the amino acids D_58_, E_72 _and K_74 _in catalysis. Surprisingly, the mutants in the MTase domain – V_356_M (AdoMet binding motif) and N_473_A (catalytic MTase motif) – also displayed a significantly reduced cleavage activity *in vitro *in comparison to the wt TspGWI protein. The double mutant V_356_M/N_473_A exhibited no detectable REase activity *in vitro*. TaqII, the closest relative of TspGWI, exhibits the "DPA**M**GTG" variant of motif I. This observation, together with the fact that TaqII REase activity is strongly stimulated by AdoMet (while TspGWI REase is slightly inhibited), points to the complex role of AdoMet in both enzymatic activities and suggests that some interaction occurs between the nuclease and MTase domains in the course of the cleavage reaction. The substitution of methionine for valine in the AdoMet-binding motif of TspGWI (**V**_356_M) could disturb AdoMet-affected communication between the nuclease domain and the MTase domain. The nature of the communication between the domains remains to be defined by further analyses.

### MTase activity of TspGWI wt and the mutant proteins

Mutant proteins were further analysed for their methylation activity. *In vitro *methylation reactions were performed using wt TspGWI enzyme and mutant proteins (D_58_A, V_356_M, N_473_A and V_356_M/N_473_A) in the DNA protection assay as described in Materials and Methods. Two types of substrate DNA were used: pBR322 plasmid DNA and the 816 bp PCR fragment. The results presented in Table 1 and Table 2 demonstrate that the mutant N_473_A (motif IV in the MTase active site) is unable to modify the TspGWI target sequence. Interestingly, the N_473_A substitution not only completely eliminates the methylation activity, but also decreases the cleavage activity of TspGWI; even so, the resulting protein retains sufficient activity to be considered a functional REase. Thus, both the REase and MTase activities of TspGWI were uncoupled, and REase-only or MTase-only TspGWI variants were obtained. This is also of high practical methodological value in gene cloning technology, allowing to use TspGWI mutants as traditional class-II enzymes for DNA cleavage or methylation. Mutants with D_58_A (nuclease active site) or V_356_M (AdoMet-binding motif I) substitutions exhibit MTase activity at the same level as the wt enzyme, despite the total or partial loss of their ability to cleave the substrate DNA. We observed that D_58_A slightly increased the modification activity (specific activity) in comparison to the wt protein (not shown). This could have been due to the absence of a competing REase activity in this variant. Interestingly, Mg^2+ ^at 10 mM concentration tested has a strong stimulatory effect on the MTase activity of the D_58_A mutant (Table 1). In the case of wt TspGWI, Mg^2+ ^is utilised for cleavage, which hinders its effect on the MTase activity. Thus, the D_58_A mutation demonstrates that the competing MTase and REase activities both share the same Mg^2+ ^cofactor. However, it cannot be ruled out that the presence of divalent cations stabilizes not perfectly folded mutant protein TspGWI, thus contributing non-specifically to retaining their enzymatic activities. Taken together with the observed inhibition of the TspGWI REase activity by AdoMet, our results show that the DNA methylation and cleavage activities of TspGWI are closely intertwined, but autonomous enough to have their functions separated.

## Conclusion

(*i*) The *tspgwi *gene coding for 120 kDa TspGWI has been sequenced and cloned.

(*ii*) Active bifunctional REase-MTase protein has been expressed in *E. coli *and purified.

(*iii*) Bioinformatics studies predicted REase and MTase binding/catalytic motifs: PD-(D/E)XK, DPAVGTG, NPPY and showed modular structure of TspGWI.

(*iv*) Series of mutants in the predicted motifs were constructed, yielding REase-deficient and MTase-deficient enzymes. Uncoupled REase and MTase activities exhibited high autonomy.

(*v*) Mutants more suitable than native TspGWI for gene cloning methodology were constructed. The cleavage and modification functions were separated into distinct proteins, while maintaining enzymatic activity, thus allowing practical use of TspGWI *in vitro *as a regular class II enzyme.

## Methods

### Bacterial strains, plasmids, media and reagents

*Thermus sp*. GW was obtained from Piotr Skowron's collection. The optimum cultivation conditions were a temperature of 60°C in a modified Luria broth (0.5% tryptose; 0.3% yeast extract; 0.2% NaCl; 0.001% dilution of 2.1 g/L stock Nitsch's trace elements; pH 7.2). The bacteria were harvested at OD_600 _= 1.6. *E. coli *DH11S {*mcrA *Δ[*mrr*-*hsdRMS*(r_K-_, m_K+_)-*mcrBC*] Δ(*lac-proAB*) Δ(*recA1398*) *deoR*, *rpsL*, *srl-thi*, *supE*/F' *proAB*^+ ^*lacI*^Q^*Z*Δ*M15*} (Life Technologies, Gaithersburg, Maryland, USA) was used for transformation of ligation mixtures and DNA propagation. Bacteria were grown in 2xYT medium [39]. For protein expression bacteria were cultivated in TB medium [39]. Media were supplemented with chloramphenicol (40 μg/ml) and 0.2% maltose. Difco media components were obtained from Becton-Dickinson (Franklin Lakes, New Jersey, USA), agarose GTG from FMC (Rockland, Massachusetts), phosphocellulose P11 resin from Whatman (Springfield Mill, UK) and hydroxyapatite HTP from Bio-Rad (Hercules, California, USA). Other chromatographic resins were from Pharmacia Biotech AB (Uppsala, Sweden). Immobilised TPCK-trypsin and the BCA Protein Assay Reagent Kit were supplied by Pierce (Rockford, Illinois, USA). Protein standards were from Pharmacia Biotech AB (Uppsala, Sweden), Sigma-Aldrich (St Louis, Missouri, USA) and New England Biolabs (Ipswich, Massachuesetts, USA). All other reagents were from Amresco (Solon, Ohio, USA) or Sigma-Aldrich, of the highest available purity. The cloning vectors pAPS (Cm^R^, MCS, col E1 *ori*, *f*1 *ori*, P_lac _and T7 promoters) and pRZ4737 (Cm^R^, P15A *ori*, *f*1 *ori*, P_R _promoter) were from Piotr Skowron's collection (pRZ4737 originally obtained from Bill Resnikoff and further modified). Plasmids pBR322 and pUC19, DNA purification kits, SmaI and SalI REases, T4 DNA ligase, T4 DNA polymerase, proofreading Taq DNA Polymerases blend (OptiTaq) and lambda DNA were from EURx Ltd. (Gdansk, Poland). BspHI REase was from New England Biolabs.

### Native TspGWI purification

The native TspGWI enzyme was isolated from *Thermus *sp. GW as described previously [10] with the additional step of size exclusion chromatography on a Sephadex G-120 column added at the end of the established procedure.

### Molecular size estimations

#### Gel electrophoresis

Protein electrophoresis under denaturating conditions was carried out on 6–10% (w/v) polyacrylamide gels in the presence of 1% (w/v) sodium dodecyl sulphate (SDS/PAGE) as described [39,40]. Protein bands were visualised by staining with 0.1% (w/v) Coomassie Brilliant Blue R-250. DNA was subjected to agarose electrophoresis on 1–2% gels and stained with ethidium bromide [39].

#### Gel filtration

Size-exclusion chromatography on a Sephadex G-120 column in buffer F (20 mM Tris-HCl pH 8.3; 3 mM MgCl_2_; 25 mM (NH_4_)_2_SO_4_; 25 mM KCl; 0.5 mM DTT; 0.05% Tween 20; 5% glycerol) was used to determine the molecular size of the purified TspGWI enzyme. Myoglobin (17 kDa), carbonic anhydrase (30 kDa), chicken ovalbumin (44 kDa), bovine serum albumin (66,4 kDa) and alcohol dehydrogenase (150 kDa) were used as standard molecular size markers.

### Proteolysis of TspGWI and amino acid sequence determination

Purified native TspGWI was subjected to limited TPCK-trypsin digestion to obtain internal polypeptides for N-terminal amino acid sequencing. Proteolysis of TspGWI was conducted in buffer T (20 mM Tris-HCl pH 8.3, 25 mM KCl, 3 mM MgCl_2_, 5% glycerol, 0.05% Tween 20, 0.5 mM DTT) with gel-immobilised TPCK-trypsin with gentle shaking at 24°C for 3 hours. The immobilised TPCK-trypsin was removed by centrifugation. The supernatant, containing TspGWI protein fragments was run on 10% SDS/PAGE denaturing gel and electroblotted onto a PVDF membrane in 100 mM CAPS-NaOH buffer pH 11.0. The N-terminal amino acid sequence analysis of blotted polypeptides was performed on a gas-phase sequencer (Model 491, Perkin Elmer-Applied Biosystems). The phenylthiohydantoin derivatives were analysed by on-line gradient high performance liquid chromatography on a Microgradient Delivery System Model 140C equipped with a Programmable Absorbance Detector Model 785A and Procise software (Perkin Elmer-Applied Biosystems).

### Determination the nucleotide sequence and cloning of the *tspgwi *gene

The gene nucleotide sequence was obtained using combination of PCR methods employing degenerated and non-degenerated primers. Amplification of the inner *tspgwi *fragment was performed with primers designed using internal amino acid sequences of tryptic peptides of TspGWI. The obtained DNA fragment was used as a sequencing start point. Subsequently, PCR with non-degenerated primers and cassette-ligation-PCR were utilized to complete the entire contig.

In the first step the forward primer: 5'-GGCCCCATCGGCGG(CG)GG(CG)GG(CG)AGCCCCGA(GA)-3' and the reverse primer: 5'-CTCCTGCGCCAGGCGCTCGTAGAAGAA-3' were used. PCR was performed using an Applied Biosystems 2720 thermocycler in 100 μl of a reaction mixture containing 10 mM Tris-HCl pH 9.1, 50 mM KCl, 1.5 mM MgCl_2_, 0.1% Triton X-100, 6% formamide, 100 ng *Thermus *sp. GW genomic DNA, 0.5 μM of each primer, 80 μM of each dNTP and 5 U Taq-Pfu DNA Polymerase blend. The cycling conditions were as follows: an initial denaturation step of 3 min at 97°C, followed by the addition of the Taq-Pfu DNA Polymerases blend at 85°C and 30 cycles of 30 s denaturation at 95°C, 30 s annealing at 55°C and 2 min elongation at 72°C. The PCR product of app. 1300 bp in length was isolated from agarose gel and cloned into the pAPS vector at the SmaI site and sequenced.

#### Cassette ligation mediated PCR

The flanking regions of the cloned TspGWI 1300 bp DNA fragment were amplified using a cassette ligation-mediated PCR approach. Cassettes were constructed by digestion of pBR322 plasmid DNA with BsuTUI/SphI or BspHI REase, respectively. DNA fragments of pBR322 of 544 bp or 539 bp in length were isolated from agarose gel and purified. Two samples of *Thermus *sp. GW genomic DNA were digested separately with TaqI or NcoI REases. The digested DNA in each sample was ligated to a compatible DNA cassette. The resulting ligation products were used as a template for PCR amplification. The nested PCR was performed using two pairs of primers.

Two primers 5'-CCATCGGTGATGTCGGCGATATAG-3' and 5'-GCTTCGCTACTTGGAGCCACTATC-3' were designed to be annealed to the cassettes, and two additional primers 5'-TCGAGGAGGCGGGTGATGAGAG-3' and 5'-GCAAGGCCCGGGGGAAATGGAT-3' were designed to be annealed to the TspGWI 1300 bp DNA fragment close to the TaqI and NcoI restriction sites, respectively. The resulting PCR products were cloned into a pAPS vector and sequenced with pAPS universal sequencing primers or insert-specific primers. Using this method three additional *tspgwi *gene fragments were identified and sequenced which, together with the internal 1300 bp *tspgwi *fragment, assembled a contig covering the complete *tspgwi *gene. Uncertain regions of the *tspgwi *gene were then re-sequenced directly from genomic DNA.

#### Analysis of nucleotide sequences

DNA sequences of the double-stranded plasmid DNA and genomic DNA were sequenced using the ABI Prism 310 automated sequencer with the ABI Prism BigDye Terminator Cycle Sequencing Ready Reaction Kit (Perkin Elmer Applied Biosystems, Foster City, CA, USA). The obtained sequence data were analysed using ABI Chromas 1.45 software (Perkin Elmer Applied Biosystems) and DNASIS 2.5 software (Hitachi Software, San Bruno, CA, USA).

The approach to obtain overexpression of the TspGWI enzyme employed the modified vector pRZ4737, a derivative of pACYC184 plasmid [41], carrying a lambda DNA section, and containing the P_R _promoter under the control of the CI repressor. The *cI *gene was located on the pRZ4737 backbone, allowing for host-independent expression in *E. coli*.

The BspHI-cleaved pRZ4737 vector was treated with T4 DNA polymerase in the presence of dNTPs [39], which created a blunt-ended vector with its ATG codon exposed for fusion. The vector thus prepared was further digested with SalI REase, treated with calf intestine alkaline phosphatase [39] and agarose gel purified. The *tspgwi *gene was PCR amplified with proofreading Taq DNA polymerase blend using the oligonucleotides 5'-ATGCCGAACTGGAGCCGTACCTAGACGA-3' and 5'-GCCTGGTCGACCTCCTCCGTGCTGAAGAC-3', which generated another ATG codon at the 5'-end introducing the SalI recognition site (underlined) after the *tspgwi *gene STOP codon at the 3'-terminus. The PCR fragment obtained was digested with SalI REase and treated with T4 polynucleotide kinase. The lineared vector DNA and restriction fragments were isolated from agarose gel [39]. The purified PCR fragment of 3450 bp was inserted directly into the expression plasmid pRZ4737 to form a pRZ-TspGWI clone. The plasmid pRZ-TspGWI was transformed into *E. coli *DH11S competent cells and plated onto 2xYT medium supplemented with chloramphenicol (40 μg/ml) and 0.2% maltose at 28°C. To ensure accuracy of the *tspgwi *gene sequence obtained, the entire assembled contig sequence was amplified from genomic *Thermus *sp. GW with proofreading polymerase and both strands re-sequenced.

### Site-directed mutagenesis

Site-directed mutagenesis within the *tspgwi *gene was carried out by PCR amplification of the entire plasmid, with changes introduced on the primers used (Table 3). The PCR reaction was performed with the proofreading Taq DNA polymerase, used within a concentration range from 1 to 5 units per reaction. The cycling conditions included an initial denaturation step of 30 s at 95°C, followed by addition of the polymerase at 85°C and 18 cycles of 30 s denaturation at 95°C, 1 min annealing at 68°C and 22 min elongation at 68°C. The final incubation for 7 min at 68°C was performed to complete the extension of PCR products. All pairs of PCR primers were also designed for the introduction of new, unique restriction sites, which did not change the coded amino acids, so as to enable an additional selection stage of mutated DNAs prior to final analysis by sequencing (Table 3). The mutant clones obtained were sequenced to verify the presence of the desired change and to ensure that no unwanted changes have been made.

**Table 3:** PCR primers used for site-directed mutagenesis.

**Primer sequence**	**Selection**	**Mutant**
5'-ACCGGCCTGGGCCGGCC**CG(CA)G**ATGGCGGTCTACCAC-3'	**BstUI** (mutant D_58_A)	**D**_58_**A** **D**_58_**E**
		
5'-GGTGGTAGACCGCCAT**C(TG)CG**GGCCGGCCCAGGCCGG-3'		

5'-TCCTGGTGGGCTTCGTG**(ACGT)(ACGT)(ACGT)**CTCAAGGCTCCGGGCAAG-3'	**SacI** restriction site disappears	**E**_72_**X**
		
5'-CTTGCCCGGAGCCTTGAG**(ACGT)(ACGT)(ACGT)**CACGAAGCCCACCAGGA-3'		

5'-CTGGTGGGCTTCGTGGAGCTC**(GC)(GC)**GGCCCC**G**GGCAGG-3'	**SmaI/ApaI** (K_74_G, K_74_R)	**K**_74_**G** **K**_74_**A** **K**_74_**R** **K**_74_**P**
		
5'-CTTGCCCGGGGCC**(GC)(GC)**GAGCTCCACGAAGCCCACAGG-3'		

5'-GTTACCGTGATAGACCCGGCC**A**TGGGCACGGGGACCTT-3'	**NcoI** (V_356_M)	**V**_356_**M**
		
5'-AAGGTCCCCGTGCCCA**T**GGCCGGGTCTATCACGGTAAC-3'		

5'-TGGTGATCCTTGGC**GCC**CCCCCCTACGACCGGGTG-3'	**NarI** (N_473_A)	**N**_473_**A**
		
5'-CACCCGGTCGTAGGGGGG**GGC**GCCAAGGATCACCA-3'		

### PCR substrates for TspGWI and TaqII

PCR substrates for TspGWI and TaqII cleavage were prepared as either containing isolated sites for both enzymes (200 bp PCR product) and double divergent sites (816 bp PCR product) or double convergent sites for TspGWI (390 bp PCR product). The 200 bp substrate was amplified from TP-84 bacteriophage DNA using primers: 5'-ATTAATAGGGACAGTTGGGGG-3' and 5'-TGAAGAGCGGTGCTGAAGG-3'. A complete digestion of 200 bp substrate with TspGWI would yield 51 bp and 147 bp fragments, while digestion with TaqII results in 63 bp and 135 bp fragments. The 816 bp PCR substrate was obtained using synthetic bovine deoxyribonuclease gene as a template, using primers: 5'-GGGCCATGGAATAGATTCC-3' and 5'-GGTCAGGGTTACCTCAACC-3'. The complete digestion of 816 bp substrate with TspGWI results in 276 bp, 68 bp and 468 bp fragments. The 390 bp PCR substrate was obtained using pBR322 plasmid DNA as a template, using primers: 5'-CTCGACCTGAATGGAAGCCG-3' and 5'-GGTGCAGGGCGCTGACTTCC-3'. A complete digestion of 390 bp substrate with TspGWI would yield 48 bp, 56 bp and 282 bp.

### Expression of TspGWI RM under P_R _promoter in *E. coli*

Clones of native and mutant TspGWI were analysed with SalI REase for the presence of the insert. Positive clones were confirmed by nucleotide sequencing. The resulting pRZ-TspGWI clone and mutants were subjected to protein expression experiments. *E. coli *DH11S was transformed with pRZ-TspGWI and mini-scale expression was performed by cultivation in 50 ml TB media supplemented with chloramphenicol and maltose at 28°C with vigorous aeration, followed by P_R _promoter induction by a temperature shift to 42°C, when OD_600 _reached 0.7. The culture growth was continued for 12 hours at 42°C. Uninduced control and induced cells were subjected to SDS/PAGE, and gels were analysed for the appearance of the expected band size of app. 120 kDa and for endonucleolytic activity in crude lysates.

### Endonuclease and methyltransferase assays

REase activity was assayed in 30 μl TspGWI digestion buffer (50 mM Tris-HCl pH 8.0 at 25°C; 10 mM MgCl_2_; 10 mM DTT) containing 1 μg of pBR322 plasmid DNA. Increasing amounts (0.2, 0.6, 1.8 μl) of chromatographic fractions or 1–2 μl of crude lysates were added to the reaction mixture and incubated for 15–60 min at 65°C. One unit of the endonuclease is defined as the amount of enzyme required to hydrolyse 1 μg of pBR322 in 1 h at 65°C, resulting in a stable partial cleavage pattern.

The *in vitro *modification activity of TspGWI enzyme was tested by the DNA protection assay, where 1 μg of pBR322 plasmid DNA was used as a substrate in 100 μl of TspGWI reaction buffer (50 mM Tris-HCl pH 8.0; 10 mM CaCl_2_; 10 mM DTT), supplemented with 200 μM AdoMet. In this buffer, devoid of Mg^2+^, only the methylation activity of the TspGWI bifunctional enzyme becomes apparent. After addition of TspGWI protein, the reaction mixture was incubated for 3 h at 65°C. Proteinase K was added to the solution and the incubation was continued for an additional 60 min at 55°C. Samples were purified to remove all traces of proteins and divalent cations from the methylation reaction mixture, and the resulting DNA was challenged with an excess of TspGWI (10 u) for 1 h in 50 μl of TspGWI reaction buffer at 65°C. The reaction products were then resolved by agarose gel electrophoresis. One unit of modification activity is defined as the amount of TspGWI methylation activity which, during 1 h at 65°C, renders 1 μg of pBR322 plasmid DNA completely resistant to cleavage by TspGWI endonucleolytic activity.

### Purification of recombinant TspGWI RM enzyme

Expression of TspGWI in *E. coli *DH11S [pRZ-TspGWI] was initiated with bacteria inoculum washed out from a Petri dish into 1 L of rich TB media, supplemented with chloramphenicol at 28°C. The culture was grown with vigorous aeration until OD_600 _reached 0.3; then the culture was transferred to a fermentor vessel containing 3 L of the media and grown until OD_600 _was 0.7. Induction was achieved with a rapid temperature increase to 42°C by the addition of 3.5 L of the medium warmed to 56°C, and growth was continued for 17 hours at 42°C. Having achieved an OD_600 _of 4.4, the culture was cooled down to 4°C and the cells were recovered by centrifugation. A bacterial pellet was suspended in cold (4°C) buffer A1 (50 mM Tris-HCl pH 7.0; 150 mM NaCl; 5 mM EDTA; 5 mM βME; 1 mM AEBSF and 20 μg/ml benzamidine) and lysozyme was added to a concentration of 0.5 mg/ml. The suspension was incubated for 30 min at 4°C and sonicated. Bacterial debris was spun down and the supernatant heated for 30 min at 70°C. The denatured proteins were removed by centrifugation. Polyethyleneimine (PEI) removal of nucleic acids and ammonium sulphate (AmS) fractionation was performed as described for the native enzyme [10]. The recombinant TspGWI protein was further purified by phosphocellulose chromatography. Peletted TspGWI was dissolved in buffer B1 (20 mM K/PO_4 _pH 7.5; 100 mM NaCl; 0.01% Triton X100, 0,5 mM EDTA; 5 mM βME, 1 mM AEBSF, 20 μg/ml benzamidine), dialysed against buffer B1 and adsorbed into phosphocellulose P11. The column was washed with buffer B1 and eluted with a gradient of 100 mM to 800 mM NaCl in buffer B1. Fractions containing TspGWI were determined by SDS/PAGE analysis and endonuclease assay. Pooled active fractions were dialysed against buffer C (20 mM Tris-HCl pH 7.0; 50 mM NaCl; 0.01% Triton X100, 1 mM EDTA; 5% glycerol, 5 mM βME, 1 mM AEBSF, 20 μg/ml benzamidine) and loaded onto Heparin-Agarose column. The column was washed with buffer C and eluted with a gradient of 50 mM to 1000 mM KCl in buffer C. Fractions containing TspGWI were determined by SDS/PAGE analysis and endonuclease assay, concentrated using YM-100 Centricons and dialysed against buffer F (20 mM Tris-HCl pH 8.3; 40 mM (NH_4_)_2_SO_4_; 25 mM KCl; 0.1 mM EDTA; 0.05% Tween 20; 0.5 mM DTT; 50% glycerol). The dialysed TspGWI preparation was stored at -20°C.

### Bioinformatic analyses

Sequence searches of the REBASE database of RM systems were done with the BLAST utility at the REBASE website [5]. Further searches of the GenBank database were carried out at NCBI with PSI-BLAST [42] with a conservative e-value threshold of 1E-30. Multiple sequence alignment of TspGWI homologues retrieved from the GenBank was calculated with MUSCLE [43]. Identification of homology between TspGWI and proteins with known structures was carried out using a new version  of the GeneSilico MetaServer [23], which is a gateway for a variety of bioinformatic methods, in particular those for the prediction of secondary structure and structurally disordered regions, and also for protein fold-recognition (FR) analysis (an attempt to match the query sequence to known protein structures). Previously, this method has been successfully used to predict structures of several nucleases, for which subsequent crystallographic analyses confirmed the validity of prediction [44-46]. Alignments between the TspGWI sequence (or its fragments) and sequences of proteins of known structure reported by FR methods were compared, evaluated, and ranked by the PCONS method [47]. Preliminary structural modelling of individual domains was carried out using the FRankenstein's Monster method [48,49] and the resulting models were evaluated with MetaMQAP [50].

## Authors' contributions

AZS performed the experiments and participated in the design and interpretation of all experimental analyses, preparation of the figures and writing of the manuscript. JMB carried out sequence analysis and bioinformatic studies, prepared figures 3 and 4, wrote bioinformatic section and helped to draft the manuscript. PMS conceived of the project, came up with the concept of the new *Thermus *sp. enzyme family, participated in the design and interpretation of experiments, coordinated its execution and drafted the manuscript. All authors read and approved the final manuscript.

## Supplementary Material

Additional file 1 ***Tspgwi *gene and its flanking regions**. The DNA sequence and the predicted amino acid sequence of the 120.2 kDa TspGWI protein is indicated in capital letters. The DNA sequences of flanking regions are indicated in italics. The internal amino acid sequences of the TspGWI enzyme, determined by chemical analysis of proteolytic fragments, are underlined. The ATG start codon, TGA and TAG stop codons are bold and underlined. The potential TspGWI Ribosome Binding Sites (RBS) are bold italics and underlined. The arrows indicate location and orientation of the TspGWI recognition sequences. Click here for file
